# S-Allylmercapto-N-Acetylcysteine (ASSNAC) Attenuates Osteoporosis in Ovariectomized (OVX) Mice

**DOI:** 10.3390/antiox13040474

**Published:** 2024-04-17

**Authors:** Itay Bleichman, Sahar Hiram-Bab, Yankel Gabet, Naphtali Savion

**Affiliations:** 1 Department of Human Molecular Genetics and Biochemistry and Goldschleger Eye Research Institute, School of Medicine, Faculty of Medical and Health Sciences, Tel Aviv University, Tel-Aviv 6997801, Israel; itayh1@mail.tau.ac.il; 2 Department of Anatomy and Anthropology, School of Medicine, Faculty of Medical and Health Sciences, Tel Aviv University, Tel-Aviv 6997801, Israel; saharari@tauex.tau.ac.il (S.H.-B.); yankel@tauex.tau.ac.il (Y.G.)

**Keywords:** osteoporosis, ASSNAC, Nrf-2, sex hormone deficiency, oxidative stress, bisphosphonates

## Abstract

Osteoporosis is a bone-debilitating disease, demonstrating a higher prevalence in post-menopausal women due to estrogen deprivation. One of the main mechanisms underlying menopause-related bone loss is oxidative stress. *S*-allylmercapto-*N*-acetylcysteine (ASSNAC) is a nuclear factor erythroid 2-related factor 2 (Nrf2) activator and cysteine supplier, previously shown to have anti-oxidation protective effects in cultured cells and animal models. Here, we studied the therapeutic potential of ASSNAC with and without Alendronate in ovariectomized (OVX) female mice. The experimental outcome included (i) femur and L3 lumbar vertebra morphometry via Micro-Computed Tomography (μCT); (ii) bone remodeling (formation vs. resorption); and (iii) oxidative stress markers in bone marrow (BM) cells. Four weeks after OVX, there was a significant bone loss that remained evident after 8 weeks, as demonstrated via µCT in the femur (cortical and trabecular bone compartments) and vertebra (trabecular bone). ASSNAC at a dose of 50 mg/Kg/day prevented bone loss after the four-week treatment but had no significant effect after 8 weeks, while ASSNAC at a dose of 20 mg/Kg/day significantly protected against bone loss after 8 weeks of treatment. Alendronate prevented ovariectomy-induced bone loss, and combining it with ASSNAC further augmented this effect. OVX mice demonstrated high serum levels of both C-terminal cross-linked telopeptides of type I collagen (CTX) (bone resorption) and procollagen I N-terminal propeptide (P1NP) (bone formation) after 2 weeks, and these returned to control levels after 8 weeks. Alendronate, ASSNAC and their combination decreased CTX and increased P1NP. Alendronate induced oxidative stress as reflected by decreased glutathione and increased malondialdehyde (MDA) levels, and combining it with ASSNAC partially attenuated these changes. These results portray the therapeutic potential of ASSNAC for the management of post-menopausal osteoporosis. Furthermore, ASSNAC ameliorates the Alendronate-associated oxidative stress, suggesting its potential to prevent Alendronate side effects as well as improve its bone-protective effect.

## 1. Introduction

Bone tissue is constantly renewed through the delicate balance between bone resorption, performed by osteoclasts, and bone formation, performed by osteoblasts. This balance is pivotal for the maintenance of the integrity and function of the skeletal system. Normally, these two processes are coupled so that the amount of resorbed bone is replaced by the same amount of new bone, thus maintaining bone mass. In osteoporosis, there is either excessive bone resorption or inadequate bone formation, leading to bone loss and increased skeletal fragility [1,2,3]. It is more prevalent in women, and around 50% of women aged 50 and older will sustain an osteoporotic fracture during their lifetime. Therefore, osteoporosis is a major health care problem, mostly among post-menopausal women [4].

According to the FDA, ovariectomy (OVX) in rodents is a well-established experimental model as it recapitulates the bone changes observed in post-menopausal women [5,6,7]; these include an initial period of high-turnover bone loss caused by the sudden loss of estrogens, followed by a low turnover and increased bone fracture risk. This model is often used to assess the preventive potential of new therapeutic agents in osteoporosis [8,9,10].

Oxidative stress results from an imbalance between the amount of reactive oxygen species (ROS) within a cell and the cell’s ability to remove these reactive peroxides and free radicals. ROS are by-products of normal oxygen metabolism; exogenous stressors that include ionizing radiation, UV light and divergent oxidizing chemicals can induce their production [11]. ROS at low levels are important for proper signal transduction; however, an excess of ROS results in oxidative stress that may damage DNA, proteins and lipids and, thus, may compromise cell structure and viability [12]. Excess ROS is implicated in the development and progression of various age-related degenerative diseases including osteoporosis [11,13,14]. Furthermore, recent studies demonstrated the capacity of antioxidant agents acting through nuclear factor erythroid 2-related factor 2 (Nrf2) activation to protect bone tissue against osteoporotic changes. These include oltipraz [15] and monomethylfumarate [16], which successfully protect the bone microarchitecture of 1,25-dihydroxyvitamin D-deficient and OVX mice, respectively, as well as Melatonin [17] and Orientin [18], which improve bone mass in OVX rats.

The agents currently used to treat osteoporosis include both anti-resorptive and pro-bone formation agents. The anti-resorptive agents are more prevalent, and among them, bisphosphonates are usually the first line of treatment for primary osteoporosis. They are known to induce osteoclast apoptosis, thereby attenuating bone resorption [19]. The skeletal effects of Alendronate, a commonly used bisphosphonate, on OVX animals and post-menopausal women are well documented [20,21,22]. However, it is becoming evident that although these drugs are effective in stopping the resorption of bone and preventing osteoporosis in women, they have a low long-term compliance [23]. Furthermore, bisphosphonates induce oxidative stress [24], associated with decreased glutathione and increased lipid peroxidation end-product malondialdehyde (MDA) [25], which are probably contributing factors to atypical fractures and osteonecrosis of the jaw, two debilitating complications of bisphosphonate treatment [26,27,28,29,30,31]. Indeed, treating cultured oral fibroblasts with bisphosphonates has demonstrated increased ROS levels [32], while oxidative stress has been detected in patients with bisphosphonate-related osteonecrosis of the jaw, accompanied by an increased oxidized glutathione (GSSG)/glutathione (GSH) ratio [33]. Alternative strategies are therefore warranted to improve the efficacy of osteoporosis treatments and reduce their side effects.

Natural protection from the deleterious activity of ROS is achieved through cellular antioxidants such as glutathione [34] and the activation of Nrf2 [35], orchestrating antioxidant and detoxification responses to oxidative and electrophilic stress. Nrf2 interacts with its inhibitory partner, Kelch-like ECH-associating protein 1 (Keap1), forming a cytoplasmic complex that is then degraded. Keap1 contains several cysteine residues that serve as sensors of the intracellular redox state, and the oxidation of their free sulfhydryl results in the dissociation of Nrf2 from Keap1 and its translocation to the nucleus [36,37]. There, Nrf2 binds to a regulatory sequence, activating the genes encoding the antioxidant and phase 2 detoxifying enzymes, thereby regulating glutathione biosynthesis and activity, the thioredoxin pathway, superoxide dismutase and heme oxygenase-1, thus protecting the cell against oxidative stress [38]. Therefore, the free sulfhydryls on Keap1 are a potential target for the development of indirect antioxidants [11]. Exploring the role of Nrf2 in bone metabolism and osteoporosis in Nrf2-deficient mice has revealed a loss of trabecular bone mineral density in femora and a reduction in cortical bone area in vertebrae in normal and OVX mice [39]. Furthermore, an Nrf2 activator effectively attenuates bone loss in OVX mice [40]. This suggests that Nrf2 may play a role in bone metabolism, as oxidative stress is an important mediator of estrogen-deficiency bone loss.

S-allylmercapto-N-acetyl-cysteine (ASSNAC) is a conjugate of S-allylmercaptan, a hydrophobic moiety rendering the molecule tissue permeability, shown to up-regulate GSH levels [41], with N-acetylcysteine (NAC), known as a supplier of cysteine, the limited precursor in glutathione biosynthesis [42]. We have demonstrated the dual activity for ASSNAC, activating Nrf2 probably through a thiol exchange reaction with free cysteine residues on Keap1 and supplying cells with cysteine [42]. The activation of Nrf2 increases the expression of phase II detoxifying enzymes including the cystine transporter (xCT) and the enzymes synthesizing glutathione. This results in a significant increase in the glutathione cellular levels, leading to the increased resistance of endothelial [42] and nerve [43] cells, *C. elegans* [44] and ocular tissues [45] to oxidative stress. We recently demonstrated the role of ASSNAC in attenuating diabetes-associated osteoporosis in mice [46], and others reported an amelioration of pulmonary fibrosis in treated mice [47]. The present study explored the role of ASSNAC alone or in combination with Alendronate in bone metabolism in OVX mice.

## 2. Materials and Methods

### 2.1. Materials

ASSNAC was synthetized as previously described [42]. Briefly, allylthiosulfate was prepared by mixing cold sodium thiosulfate (Fluka, Buchs, Switzerland) with allyl bromide, and then, NAC (both from Sigma-Aldrich, Saint Louis, MO, USA) was added (at pH 8.0) to form ASSNAC. ASSNAC was extracted at room temperature (at pH 3.0) using t-Butyl methyl ether that was evaporated under vacuum conditions, resulting in the formation of yellowish oil crystals of ASSNAC. On HPLC, using a C:18 hydrophobic column, ASSNAC appeared as a major peak representing 96.8% of the loaded material at a retention time of 6.23 min. One contaminating peak of a less hydrophobic material was detected at a retention time of 3.91 min (which differs from the NAC retention time), representing 3.2% of the loaded material. An ASSNAC aqueous stock solution (40 mM; pH 7.4) was prepared by dissolving 100 mg of ASSNAC in 8.48 mL DDW plus 2.12 mL Na_3_PO_4_ (0.2 M); further dilutions were performed in PBS. All ASSNAC solutions were kept at 4 °C.

5-Sulfosalicylic acid (SSA), oxidized glutathione (GSSG), 2-vinylpyridine, 5,5′-dithiobis(2-nitrobenzoic acid) (DTNB), β-nicotinamide adenine dinucleotide 2′-phosphate reduced tetrasodium salt (NADPH), glutathione reductase and Alendronate were purchased from Sigma (St. Louis, MO, USA).

### 2.2. Animals

Female mice (C57BL/6J-Rcc; 10 weeks old; *n* = 161) purchased from Envigo Ltd. (Rehovot, Israel) were housed at an ambient temperature of 23 °C with a 12 h light/dark cycle and ad libitum access to water and food. The experiments were carried out in accordance with the National Institute of Health guide for the care and use of laboratory animals. The mice’s health and weight were monitored weekly and every 2 weeks, respectively. All mice displaying a 15% weight loss and inability to ambulate and access food or water were euthanized for humane reasons.

### 2.3. OVX Surgery

Mice (12 weeks old) were anesthetized using an intraperitoneal injection of 0.1 mL per 10 gr body weight of saline containing Ketamine (1.0 mg) (Vetoquinol, Lure, France) and Xylazine (0.1 mg) (Eurovet, Bladel, Netherlands). Following bilateral incisions (7 to 10 mm) made through the skin and musculature at the dorsolateral aspect of the lumbar region, the peritoneum was opened, both ovaries were accessed and either resected (OVX) or left in place (Sham operation). Muscles and skin were sutured, and Dipyrone (Vetmarket, Shoham, Israel) was administered in the drinking water (2 mg/mL) for 3 days post surgery.

### 2.4. Treatment Regimen

Based on our previous studies [43,46], ASSNAC at doses of 50 and 20 mg/kg was dissolved in PBS and injected (0.1 mL; i.p.) 5 times per week. Alendronate at a dose of 40 mg/Kg was dissolved in PBS and injected (0.1 mL; i.p.) twice a week [48]; PBS as a control treatment was injected (0.1 mL; i.p.) 5 times per week. Treatment started at the age of 12 weeks and continued up to 8 weeks. At the end of treatment, mice were sacrificed in CO_2_ atmosphere.

### 2.5. Experimental Groups

**Figure 1:**
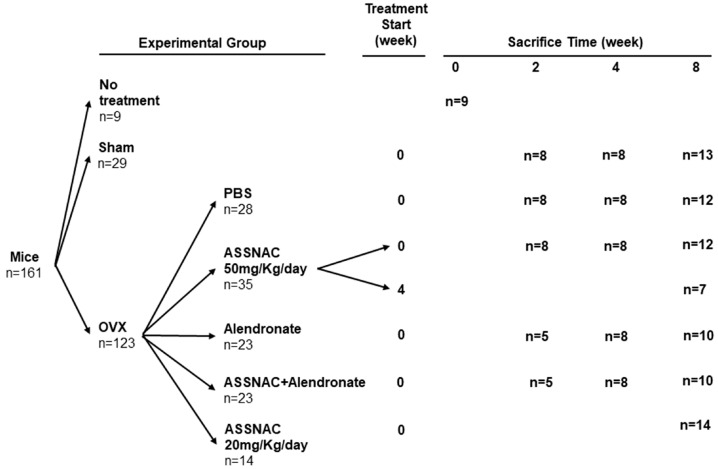
Schematic of the experiment protocol. The start and end times of each treatment are presented in the scheme. The “n” indicates the number of mice in each experimental group and time point.

Mice were randomly assigned to the treatment groups as detailed in Figure 1. Along the experimental procedure, some mice died, and some dissected femurs and vertebra were damaged and not analyzed; consequently, the actual number of analyzed femur and vertebra samples is indicated in each figure legend.

### 2.6. Femur and Vertebra Collection

Mice were killed in each experimental group at the time points indicated in Figure 1. One femur and spine (L3 vertebra) of each mouse was dissected and subjected to Micro-Computed Tomography (μCT) analysis.

### 2.7. Femur and Vertebra µCT Analysis

Dissected femurs and vertebra were fixed in paraformaldehyde solution (4%; 48 h), washed and kept in ethanol (70%) until analyzed using a µCT50 system (Scanco Medical AG, Wangen-Brüttisellen, Switzerland) as previously reported [49]. Briefly, whole femurs and L3 vertebrae were scanned at a 10 μm nominal isotropic resolution, with 90 kV energy, 88 µA intensity and 1000 projections at a 1000 msec integration time. All quantitative parameters were generated as previously described and termed according to the guidelines for the assessment of bone microstructures in rodents using μCT [50,51].

Femur trabecular bone parameters were measured in the secondary spongiosa of the distal femoral metaphysis, defined as a 3 mm height volume ending distally at the proximal-most border of the primary spongiosa (the calcified part of the growth plate). The following femur full bone parameters were analyzed: bone mineral density (Full vBMD; mg/cm^3^) and bone volume fraction (Full BV/TV; %). Cortical bone parameters were determined in a 1 mm height ring located at the mid-diaphysis and included the cortical volumetric bone mineral density (Ct.vBMD; mg/cm^3^), cortical area fraction (Ct.BA/TA; %), thickness (Ct.Th.; mm) and moment of inertia (areal MOI, mm^3^). Trabecular bone parameters included trabecular volumetric bone mineral density (Tb.vBMD; mg/cm^3^), bone volume fraction (Tb.BV/TV; %), number [Tb.N.; mm^−1^] and spacing [Tb.Sp.; mm]. In the L3 vertebra, we analyzed Tb.vBMD (mg/cm^3^), tissue mineral density (TMD; mg/cm^3^), Tb.BV/TV (%) and thickness (Tb.Th.; mm).

### 2.8. Determination of Procollagen I N-Terminal Propeptide (P1NP) and C-terminal Cross-Linked Telopeptide of Type I Collagen (CTX)

#### 2.8.1. Blood Sample Collection

Blood samples were collected from mice of the Sham surgery and OVX surgery groups treated with PBS, ASSNAC (50 mg/kg/day), Alendronate and ASSNAC plus Alendronate at time points of 0, 2 and 8 weeks (n = 4 for each point). Samples were collected between 2 P.M. and 4 P.M. after 8 h of fasting (with free access to water) from the facial sub-mandibular vein (0.4 mL). The samples were placed in Microtainer plain tubes with SST gel (ref. 365968 B50) (Becton-Dickinson, Franklin Lakes, NJ, USA) and centrifuged (9000× *g*; 6 min; 4 °C), and the obtained serum was stored at −20 °C.

#### 2.8.2. CTX and P1NP Assays

Bone formation and resorption were monitored using the serum levels of P1NP and CTX, respectively. Serum samples were measured before the OVX/Sham operations (day 0) and 2 and 8 weeks after the surgery. The CTX and P1NP were determined using RATLAPS EIA and mouse P1NP EIA kits, respectively, both purchased from IDS (Tyne & Wear, UK). The results were recorded in an Elisa Reader ELx808 (Bio-Tek Instruments Inc., Winooski, VT, USA) according to the manufacturer’s instructions.

### 2.9. Glutathione and MDA Determination

#### 2.9.1. BM Cell Collection

BM cells were extracted from one femur of each mouse of the Sham surgery and OVX surgery groups treated with PBS, ASSNAC (50 mg/kg/day), Alendronate and ASSNAC plus Alendronate at the 8-week time point. BM cells were flushed out with 1 mL PBS through the insertion of a 25 G needle into the femur. The cells were collected via centrifugation, re-suspended in 1 mL PBS, divided into two fractions of 0.75 mL and 0.25 mL and centrifuged, and the final cell pellets were stored at −20 °C and used for glutathione and MDA determination, respectively.

#### 2.9.2. Glutathione Assay

Cell pellets were lysed in HCl (10 mM) followed by freezing and thawing three times. Proteins were precipitated using 5-Sulfosalicylic acid (10%) followed by centrifugation (10,000× *g*), and supernatants were taken for glutathione (GSH + GSSG) determination as previously described [42,43], using the Anderson recycling method [52]. To determine GSSG, 2-vinylpyridine was used to conjugate GSH and remove it from the mixture as previously described [52]. Protein pellets were lysed in 0.5 N NaOH and quantified using the Lowry method [53], using BSA as a standard. Glutathione and protein values were recorded at 412 nm and 660 nm, respectively, using an ELx808 Elisa Reader (Bio-Tek Instruments Inc., Winooski, VT, USA), and the glutathione results were calculated as nmole/mg protein.

#### 2.9.3. MDA Assay

Lipid peroxidation results in the formation of Malonaldehyde bis (MDA) and its level can be determined through interaction with thiobarbituric acid forming a fluorescent product [54]. Briefly, BM cells in sodium phosphate buffer (10 mM; PH 7.2) containing butylhydroxytoluene (BHT) (0.2 mM; in 0.15% ethanol solution) and EDTA (1 mM) were lysed through 3 cycles of freezing and thawing in liquid nitrogen. Thiobarbituric acid (0.5%) was added, the mixture was heated (100 °C; 15 min), and the fluorescent product was extracted into Butanol and measured (Ex. 485 nm/Em. 528 nm) using an FLx800 Fluorescence Reader (Bio-Tek Instruments Inc., Winooski, VT, USA). The MDA results are presented as nmole/mg protein using Malonaldehyde bis + diethyl acetal as a standard curve. All reagents used in this assay purchased from Sigma (St. Louis, MO, USA).

### 2.10. Statistical Analysis

The results are presented as the mean ± SD, and statistical significance between more than two groups was evaluated using either 1- or 2-way ANOVA with Tukey’s multiple comparison test. Differences at *p* < 0.05 were considered statistically significant, and 0.10 > *p* > 0.05 was considered close to significant.

## 3. Results

### 3.1. Effect of ASSNAC and Alendronate on Femur Microarchitecture in OVX Mice

**Figure 2:**
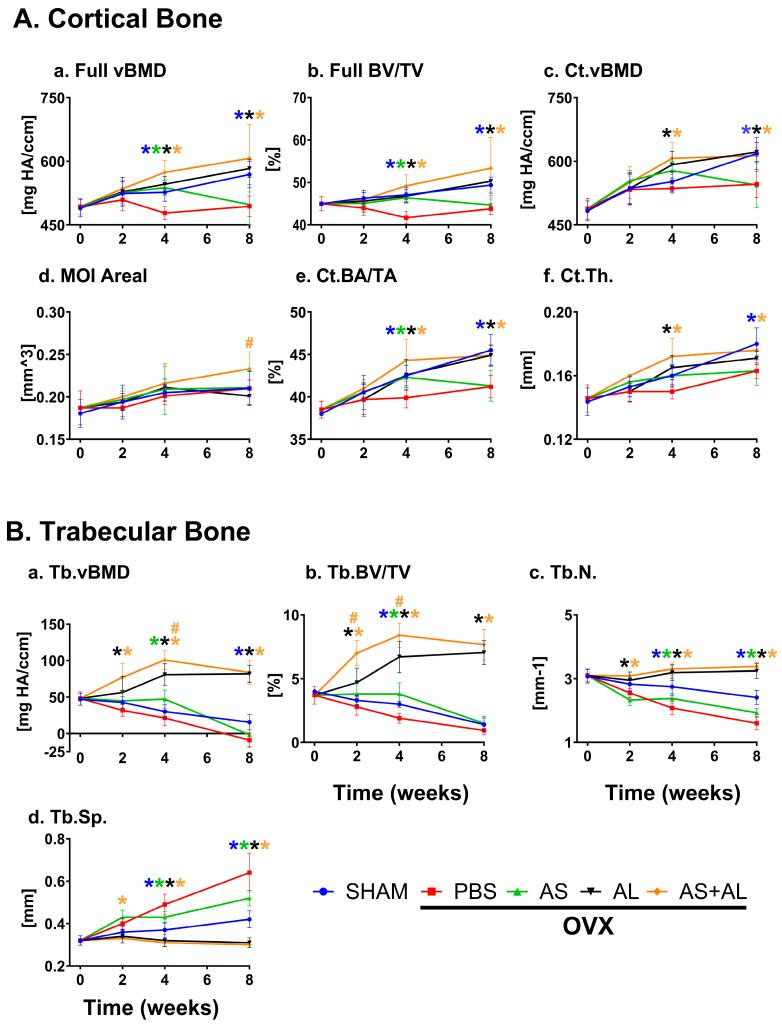
Time-dependent effect of ASSNAC and Alendronate on femur full and cortical bone parameters (**A**) and trabecular bone parameters (**B**) in OVX mice. The non-treated female mice (12 weeks old; time point, 0; n = 7); Sham-operated mice injected with PBS (blue; n = 8, 7 and 12); and OVX-operated mice injected with either PBS (red; n = 7, 7, and 11), ASSNAC (AS; 50 mg/kg, five times per week; green; n = 6, 7 and 11), Alendronate (AL; 40 mg/kg, twice a week; black; n = 5, 8 and 6) or AS + AL (orange; n = 4, 7 and 5) were sacrificed at 2, 4 or 8 weeks, respectively. Femurs were dissected and analyzed via μCT, and the following parameters were used (mean ± SD) for the cortical bone (**A**): a. full bone vBMD and b. BV/TV; c. Ct.vBMD, d. MOI Areal, e. Ct.BA/TA and f. Ct.Th. And for the trabecular bone, the following parameters were used (**B**): a. Tb.vBMD, b. Tb.BV/TV, c. Tb.N. and d. Tb.Sp. Significant differences were evaluated using two-way ANOVA with Tukey’s multiple comparison test. * Significant difference between each treatment and the OVX + PBS at the same time point (*p* < 0.05); # significant difference between AS + AL and AL at the same time point (*p* < 0.05). The color of the symbols refers to the treatment that shows significant differences.

Femurs of Sham- and OVX-operated mice, injected with PBS, Alendronate, ASSNAC or their combination, were collected before (time point: 0) and 2, 4 and 8 weeks post-OVX and subjected to µCT analysis (Figure 2).

The femurs of Sham-operated mice demonstrated a continuous time-dependent growth of full (vBMD and BV/TV) and cortical (Ct.vBMD, Ct.BA/TA and Ct.Th.) bone parameters (Figure 2A), while the trabecular bone mass (Tb.vBMD, Tb.BV/TV and Tb.N.) slightly declined (Figure 2B).

OVX mice injected with PBS demonstrated an osteoporosis-like bone mass, as compared to the Sham controls. Femurs displayed a significant decrease in full bone vBMD (−9% and −13%) and BV/TV (−11% and −11%), midshaft Ct.BA/TA (−6% and −9%) (Figure 2A) and Tb.N. (−24% and −34%) (Figure 2B) 4 and 8 weeks post ovariectomy, respectively. Furthermore, significant decreases in Ct.vBMD (−12%), Ct.Th. (−7%) (Figure 2A) and Tb.vBMD were demonstrated only at 8 weeks post ovariectomy, and in Tb.BV/TV (−33%) only at 4 weeks post ovariectomy (Figure 2B). 

Alendronate prevented the ovariectomy-induced deterioration of most bone parameters, as emphasized by the significantly higher full bone vBMD (+14%, +18%) and BV/TV (+12%, +15%) and midshaft Ct.vBMD (+10%, +14%) and Ct.BA/TA (+7%, +9%) at 4 and 8 weeks post ovariectomy, respectively, and a significant increase in Ct.Th (+7%) only at 4 weeks post ovariectomy (Figure 2A). In the distal femur, Tb.vBMD, Tb.BV/TV and Tb.N. were improved at 2, 4 and 8 weeks post ovariectomy, and Tb.Sp. was significantly reduced only at 4 and 8 weeks post ovariectomy (Figure 2B). 

ASSNAC at a dose of 50 mg/Kg/day partially prevented the deleterious effects of ovariectomy in the femurs of the treated mice. It maintained significantly higher full bone vBMD (+13%) and BV/TV (+11%), midshaft Ct.BA/TA (+6%) (Figure 2A) and trabecular Tb.vBMD and Tb.BV/TV (+98%) at 4 weeks post ovariectomy. Furthermore, the ASSNAC treatment resulted in higher Tb.N. (+14%, +20%) and lower Tb.Sp. (−12%, −18%) in the distal femur at 4 and 8 weeks post ovariectomy, respectively (Figure 2B). 

The combined treatment of Alendronate and ASSNAC significantly enhanced the protective effect of Alendronate demonstrated in femoral Tb.BV/TV (+39% and +25%) at 2 and 4 weeks post ovariectomy, respectively, and in Tb.vBMD (+24%) at 4 weeks post ovariectomy (Figure 2B). Furthermore, the combined treatment improved the midshaft MOI (+13%) at 8 weeks post ovariectomy, which was neither deteriorated in OVX mice nor improved through Alendronate alone (Figure 2A).

**Figure 3:**
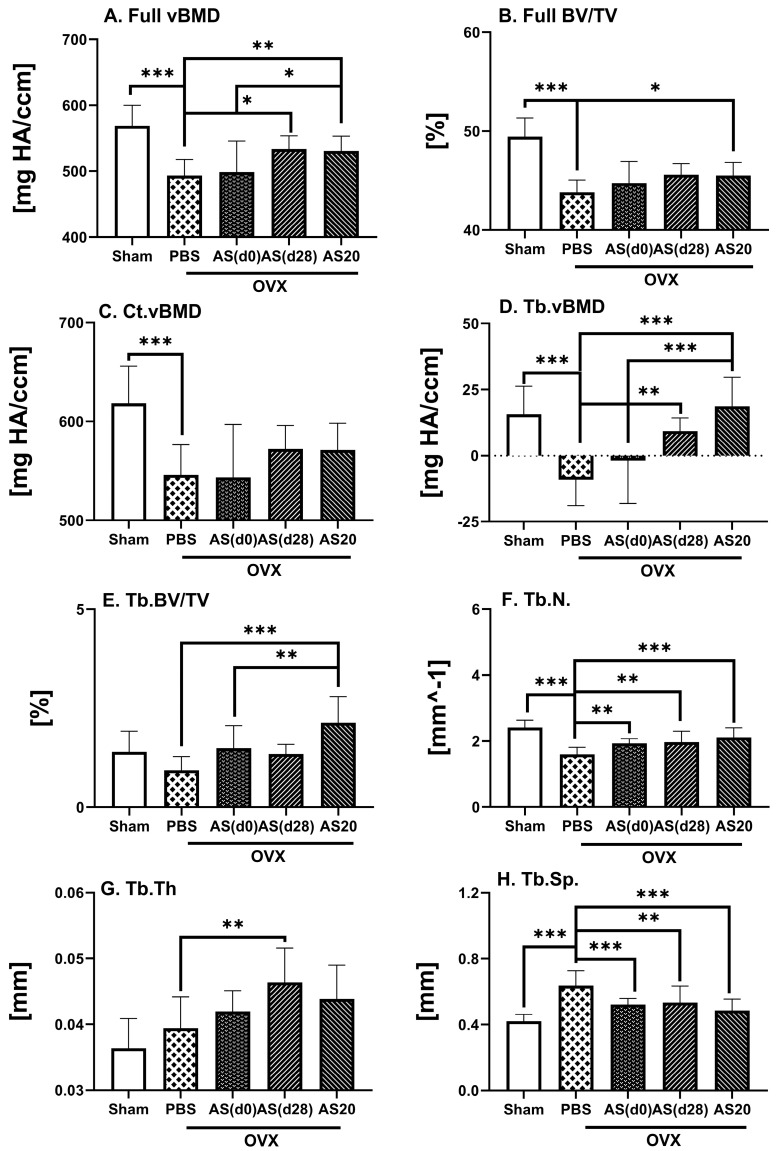
ASSNAC effect on femur microarchitecture in established osteoporosis and its effect at a lower dose in OVX mice. Female mice (12 weeks old) were Sham-operated and treated with PBS (n = 12) or ovariectomy-operated and treated with either PBS (n = 11) or ASSNAC (five time per week) at a dose of 50 mg/kg starting on day 0 for 8 weeks [AS(d0); n = 11] or on day 28 for 4 weeks [AS(d28); n = 6] or at a dose of 20 mg/kg starting on day 0 for 8 weeks (AS20; n = 14). Femurs were dissected and subjected to μCT analysis, and the following parameters are presented (mean ± SD): full bone—(**A**) Full vBMD and (**B**) Full BV/TV; cortical bone—(**C**) Ct.vBMD; trabecular bone—(**D**) Tb.vBMD, (**E**) Tb.BV/TV, (**F**) Tb.N., (**G**) Tb.Th. and (**H**) Tb.Sp. Significant differences, evaluated using one-way ANOVA with Tukey’s multiple comparison test, are presented. * 0.10 > *p* > 0.05 (close to significant); ** *p* < 0.05; *** *p* < 0.005.

An osteoporosis-like condition was already established at 4 weeks post ovariectomy; therefore, ASSNAC treatment starting at day 28 post ovariectomy [AS(d28)] would test its capacity to rescue the osteoporosis-like bone parameters. Indeed, the ASSNAC treatment started at day 28 post ovariectomy and continued for the next 4 weeks resulted in a close to significant increase in femur full bone vBMD and significant increase in trabecular Tb.vBMD, Tb.N. and Tb.Th., as well as a decrease in Tb.Sp. compared to OVX + PBS (Figure 3).

In parallel, we studied the effect of ASSNAC at a dose of 20 mg/Kg/day (AS20) compared to 50 mg/Kg/day [AS(d0)] starting immediately following ovariectomy surgery on femur microarchitecture (Figure 3). Overall, the lower dose resulted in more favorable outcomes than the higher dose for full bone vBMD [+8% vs. PBS and +6% vs. AS(d0) (close to significant)] and BV/TV [+4% vs. PBS (close to significant)], for Tb.vBMD [vs. both PBS and AS(d0)], Tb.BV/TV [+126% vs. PBS and +43% vs. AS(d0)], Tb.N. (+32% vs. PBS) and Tb.Sp. (−24% vs. PBS) (Figure 3). 

**Figure 4:**
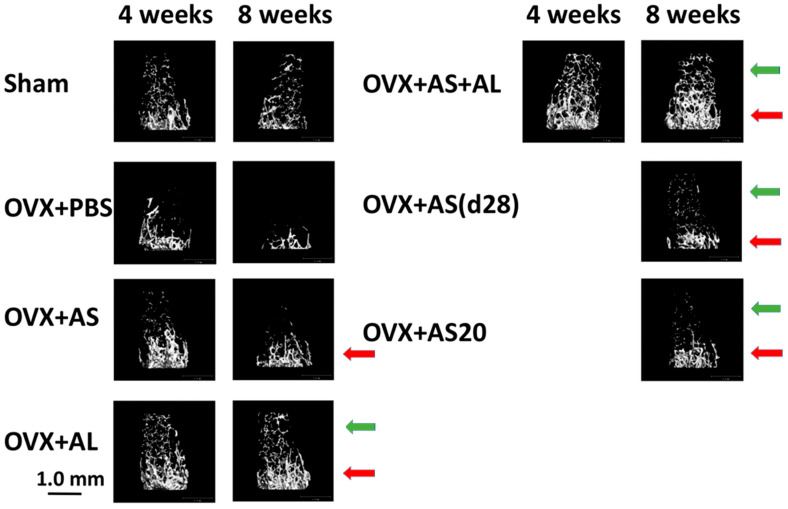
Femur bone µCT images. The µCT femur images of mice treated as described in Figure 2 (Sham, OVX + PBS, OVX + AS, OVX + AL and OVX + AS + AL) and in Figure 3 [OVX + AS(d28) and OVX + AS20] for 4 and 8 weeks are presented. The red arrows point to the image area with higher bone intensity, and the green arrows point to the appearance of some new diffused bone spots in the various treatments compared to the respective OVX + PBS image area at the 8-week time point.

The results presented in Figure 2 and Figure 3 are supported by the µCT 3D images rendering of representative femurs (Figure 4) demonstrating: (i) ovariectomy induces a decrease in bone mass resembling an osteoporosis-like process (Figure 4 OVX + PBS vs. Sham); (ii) Alendronate completely prevents bone loss in OVX mice: (Figure 4, OVX + AL vs. OVX + PBS); (iii) ASSNAC partially protects bone microarchitecture in OVX mice (Figure 4, OVX + AS vs. OVX + PBS); (iv) ASSNAC on top of Alendronate further increases bone mass compared to Alendronate alone (Figure 4, OVX + AS + AL vs. OVX + AL); (v) ASSNAC treatment starting on day 28 increases bone mass in OVX mice [Figure 4, OVX + AS(d28) vs. OVX + PBS]; and (vi) ASSNAC at a dose of 20 mg/Kg/day improved bone mass better than at a dose of 50 mg/Kg/day (Figure 4, OVX + AS20 vs. OVX + AS). Altogether, our data portray ASSNAC as an effective treatment for the restoration of femur bone mass in osteoporotic mice.

### 3.2. Effect of ASSNAC and Alendronate on Vertebra Microarchitecture in OVX Mice

**Figure 5:**
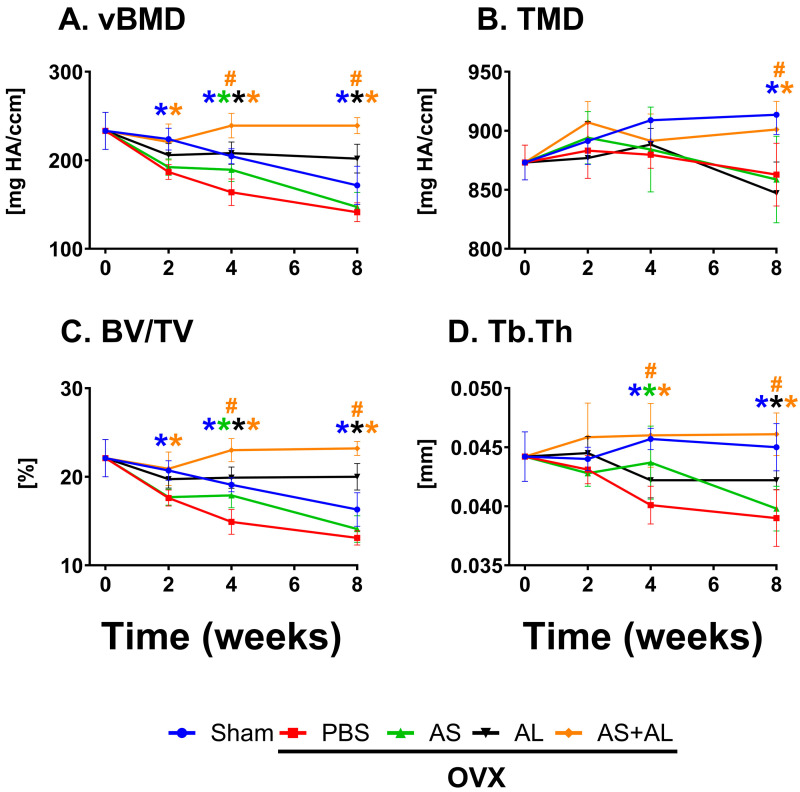
Time dependent effect of ASSNAC and Alendronate treatment on L3 vertebra microarchitecture in OVX mice. Female mice (12 weeks old) were killed (time point 0; n = 8) and Sham-operated and treated with PBS (blue; n = 8, 7 and 8) or OVX-operated and treated with either PBS (red; n = 6, 7, and 9); ASSNAC (AS; 50 mg/kg, five time per week; green; n = 7, 7 and 8); Alendronate (AL; 40 mg/kg, twice a week; black; n = 5, 8 and 9); or AS + AL (orange; n = 5, 6 and 9) for 2, 4 or 8 weeks, respectively. The L3 vertebrae were dissected and analyzed via μCT, and the following parameters are presented (mean ± SD): (**A**) vBMD, (**B**) TMD, (**C**) BV/TV and (**D**) Tb.Th. Significant differences, evaluated using two-way ANOVA with Tukey’s multiple comparison test, are presented. * Significant difference between each treatment and the OVX + PBS at each time point (*p* < 0.05); # significant difference between AS + AL and AL at each time point (*p* < 0.05). The color of the symbols refers to the treatment that shows significant difference.

**Figure 6:**
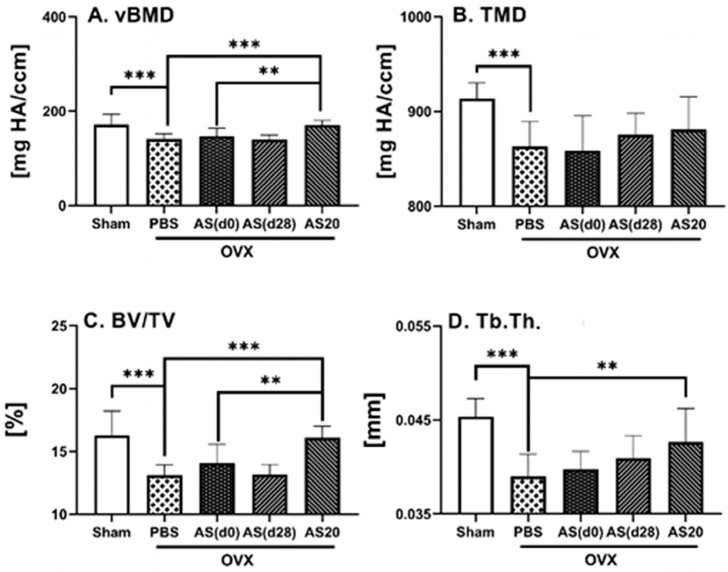
Effect of ASSNAC on L3 vertebra microarchitecture in established osteoporosis and its effect at a lower dose in OVX mice. Female mice (12 weeks old) were Sham-operated and treated with PBS (n = 8) or OVX-operated and treated with either PBS (n = 9) or ASSNAC (five time per week) at a dose of 50 mg/kg/day starting on day 0 for 8 weeks [AS(d0); n = 8] or starting on day 28 for 4 weeks [AS(d28); n = 6] or at a dose of 20 mg/kg/day (AS20; n = 9) for 8 weeks; L3 vertebrae were dissected and subjected to μCT analysis, and the following parameters are presented (mean ± SD): (**A**) vBMD, (**B**) TMD, (**C**) BV/TV and (**D**) Tb.Th. Significant differences, evaluated using one-way ANOVA with Bonferroni’s multiple comparison test, are presented. ** *p* < 0.01 and *** *p* < 0.001.

Sham-operated mice between the age of 12 and 20 weeks demonstrated age-related decreases in vertebral vBMD (−25%) and Tb.BV/TV (−12%) and a slight increase in TMD (+4%) (Figure 5). OVX mice treated with PBS, compared to Sham-operated mice, demonstrated significant deteriorations in vBMD (−17%, −20% and −18%) and Tb.BV/TV (−15%, −22% and −19%) at 2, 4 and 8 weeks post ovariectomy, respectively, Tb.Th (−12% and −14%) at 4 and 8 weeks post ovariectomy, respectively, and TMD (−6%) at 8 weeks post ovariectomy. Alendronate treatment significantly prevented the ovariectomy-induced deterioration of vBMD (+27% and +43%) and Tb.BV/TV (+34% and +52%) at 4 and 8 weeks post ovariectomy, respectively, and Tb.Th (+8%) at 8 weeks post ovariectomy. ASSNAC treatment of ovariectomy-induced bone deterioration partially attenuated this process by significantly protecting the vertebral vBMD (+15%), Tb.BV/TV (+20%) and Tb.Th. (+9%) only 4 weeks post ovariectomy(Figure 5). Such an effect was not demonstrated when ASSNAC treatment was started on day 28 [AS(d28)]; it did not prevent the deterioration of the vertebral bone microarchitecture (Figure 6). The combined treatment of Alendronate and ASSNAC significantly enhanced the protective effect of Alendronate alone on vBMD (+15% and +18%), Tb.BV/TV (+16% and +16%) and Tb.Th. (+9% and +9%) at 4 and 8 weeks post ovariectomy, respectively, and on TMD (+6%) 8 weeks post ovariectomy (Figure 5).

In the vertebra, similarly to the femur, the lower dose of ASSNAC exhibited a more prominent effect than the higher dose on vBMD [+23% vs. PBS and +17% vs. AS(d0)], Tb.BV/TV [+25% vs. PBS and +16% vs. AS(d0)] and Tb.Th. (+10% vs. PBS) (Figure 6).

### 3.3. Effect of ASSNAC and Alendronate on Collagen Metabolism in OVX Mice

**Figure 7:**
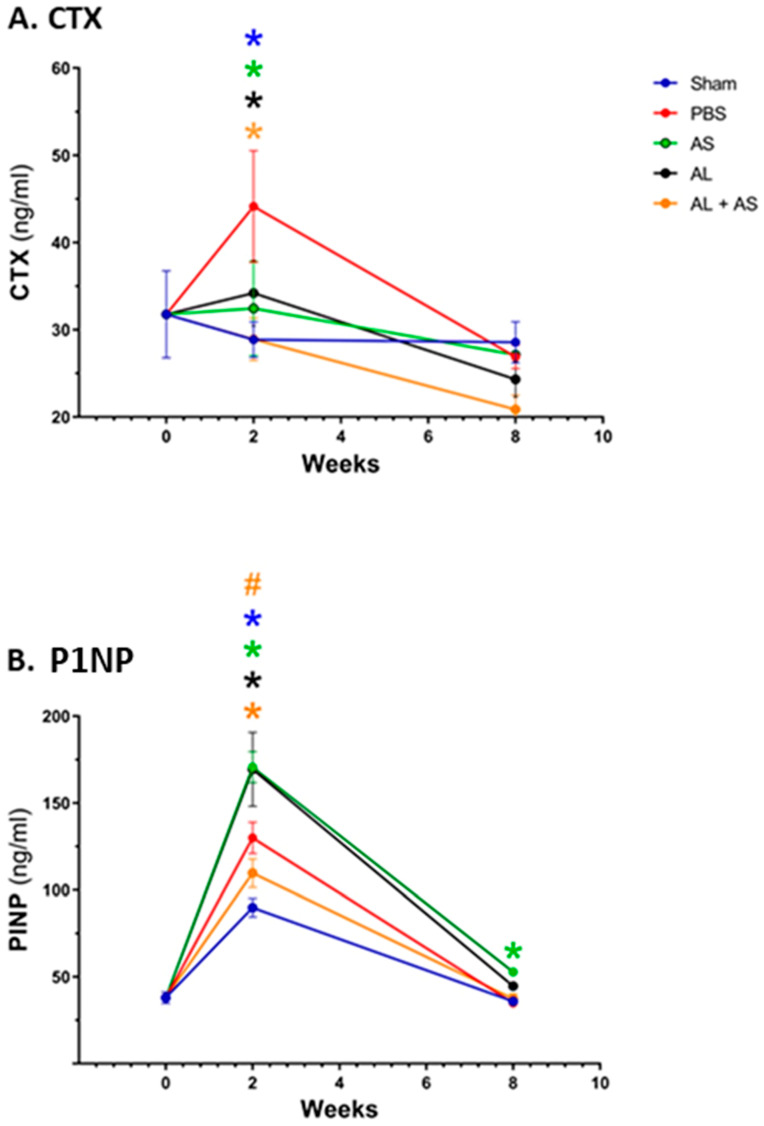
Effect of ASSNAC and Alendronate on plasma levels of CTX and P1NP. Blood samples were collected from mice treated as described in Figure 1 at time points of 0, 2 and 8 weeks, and the plasma levels of CTX (**A**) and P1NP (**B**) were determined using specific kits. The results (mean ± SD; n = 4) and significant differences, evaluated using two-way ANOVA with Tukey’s multiple comparison test are presented. * Significant difference between each treatment and the OVX + PBS at each time point (*p* < 0.01). # Significant difference between AS + AL and AL at each time point (*p* < 0.05). The colors of the symbols refer to the treatments that show a significant difference.

The CTX plasma levels of Sham-operated mice remained similar throughout the 8 weeks of the experiment (Figure 7A). In OVX mice treated with PBS, CTX plasma levels were significantly higher than in Sham-operated mice at 2 weeks post ovariectomy, while returning to the Sham-operated mice levels at 8 weeks post ovariectomy. Treatment with Alendronate, ASSNAC or their combination significantly prevented the increase in CTX levels caused by ovariectomy.

The P1NP levels of Sham-operated mice significantly increased at 2 weeks post day 0 and returned to the initial level at 8 weeks (Figure 7B). A further significant increase in P1NP levels was demonstrated in OVX mice at 2 weeks post ovariectomy, which returned to those of the Sham-operated mice at 8 weeks post ovariectomy. Both the Alendronate and ASSNAC treatments further increased the levels at 2 weeks post ovariectomy but only ASSNAC continued to maintain a slightly increased level of P1NP at 8 weeks post ovariectomy. In contrast, the treatment with ASSNAC combined with Alendronate significantly decreased P1NP levels at 2 weeks post ovariectomy.

### 3.4. Effect of ASSNAC and Alendronate on Glutathione and MDA Levels in Femur-Derived BM in OVX Mice

**Figure 8:**
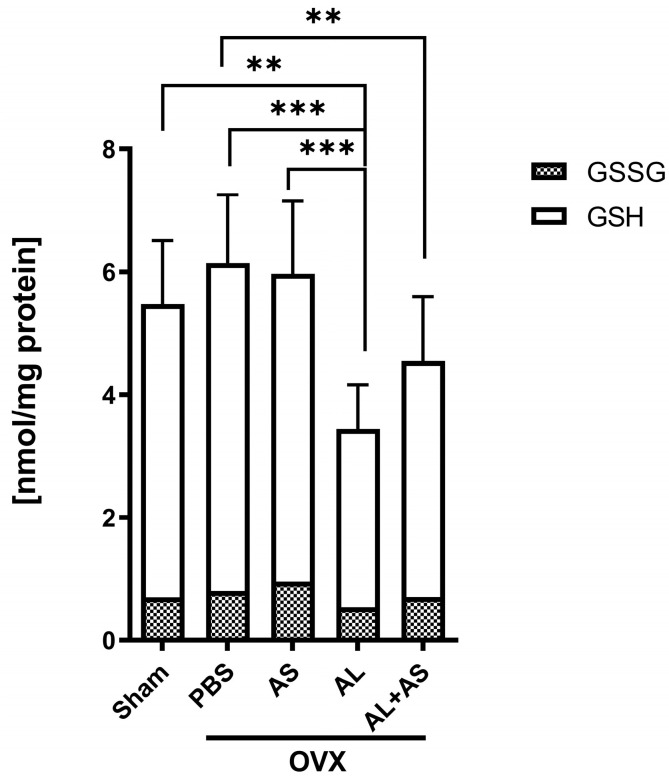
Effect of ASSNAC and Alendronate on glutathione levels in femur-derived BM cells. Mice were treated for 8 weeks as described in Figure 1, and BM cells were collected from femurs for the determination of reduced (GSH; open part of the bar) and oxidized glutathione (GSSG; dashed part of the bar). The results of the following treatments are presented (mean ± SD): Sham (n = 7) and OVX treated with PBS (n = 10), AS (n = 9), AL (n = 7), or AL + AS (n = 9). Significant differences evaluated using two-way ANOVA with Tukey’s multiple comparison test are marked with ** *p* < 0.01 and *** *p* < 0.001.

**Figure 9:**
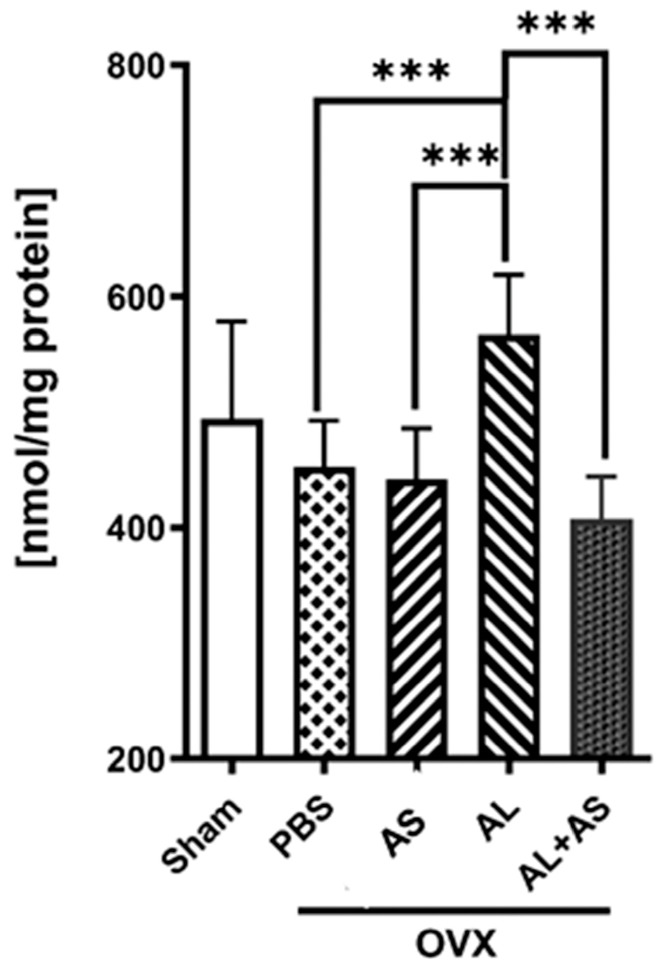
Effect of ASSNAC and Alendronate on MDA levels in femur BM cells. Mice were treated for 8 weeks as described in Figure 1, and BM cells were collected from femurs for MDA determination. The results of the following treatments are presented (mean ± SD): Sham (n = 5) and OVX treated with PBS (n = 6), AS (n = 5), AL (n = 7) or AL + AS (n = 9). Significant differences evaluated using two-way ANOVA with Tukey’s multiple comparison test are marked with *** *p* < 0. 01.

The levels of both glutathione (Figure 8) and MDA (Figure 9) did not change much from those demonstrated in OVX mice treated with PBS or ASSNAC. The Alendronate treatment significantly reduced the glutathione levels (−45%) and increased the MDA levels (+47%). However, the addition of ASSNAC on top of Alendronate resulted in a significant decrease in MDA (−46%) and a non-significant increase in glutathione levels (+32%) compared to Alendronate alone. The level of oxidized glutathione (GSSG) was low in all samples in the range of 13–15% of the total glutathione with no difference between the groups (Figure 8).

## 4. Discussion

In this study, we explored the potential of ASSNAC alone or in combination with Alendronate to attenuate the development of osteoporosis as well as to affect the BM cells’ oxidative state in OVX mice. The results show the therapeutic potential of ASSNAC alone or in combination with Alendronate in attenuating osteoporosis and the role of ASSNAC in ameliorating the Alendronate-associated oxidative stress.

OVX mice treated with PBS demonstrated deteriorations in both femur and vertebra bone microarchitecture, characteristic of osteoporosis. Femur deterioration was evident in the full bone (vBMD and BV/TV), cortical bone (Ct.vBMD, Ct.BA/TA and Ct.Th.) and trabecular bone (Tb.vBMD, Tb.BV/TV, Tb.N. and Tb.Sp.) parameters, while in vertebrae, it was evident in vBMD, TMD, BV/TV and Tb.Th. As expected, the control drug, Alendronate, inhibited bone resorption and prevented the deterioration of all femur and vertebra parameters except for vertebra TMD.

ASSNAC treatment at a dose of 50 mg/Kg/day significantly prevented the deterioration of bone microarchitecture as evident in femur full vBMD and BV/TV, cortical Ct.BA/TA and all trabecular and vertebra parameters except TMD at 4 weeks post ovariectomy, but only in femur trabecular Tb.N. and Tb.Sp. parameters at 8 weeks post-ovariectomy. The more pronounced effect of ASSNAC at the 4-week versus the 8-week post-ovariectomy time point suggests that ASSNAC significantly prevented the ovariectomy-associated deteriorations in femur and vertebra bone during the first 4 weeks of treatment, a period characterized by high-turnover bone loss.

ASSNAC treatment for 4 weeks starting on day 28 post ovariectomy demonstrated a significant restoration of femur trabecular parameters but not full bone and cortical or any of the vertebra parameters, suggesting that ASSNAC may partially restore the ovariectomy-associated deterioration of femur but not of vertebra bone.

In a previous study, we demonstrated the protective effect of ASSNAC on diabetes-associated osteoporosis; we showed that ASSNAC restored Tb.BV/TV and the Tb.N in the distal femur of db/db mice [46]. The current study demonstrated the significant protective effect of ASSNAC on both the appendicular and axial skeleton in a model of post-menopausal osteoporosis.

Previous studies with antioxidant compounds such as the peptides PIISVYWK and FSVVPSPK from *Mytilus edulis* [55], the adipokine Apelin-13 [56], Ginkgolide B from Ginkgo biloba [57], polyphenolic extract from melon (*Cucumis melo* L.) [58], Orientin, a C-glycosyl flavonoid [18], and Resveratrol, a polyphenolic compound from grapes [59], demonstrated a protective effect on the bone microarchitecture of OVX mice or rats similarly to that of ASSNAC. Furthermore, the antioxidants Corylifol A [60] and lycopene [61] were shown to target the femur trabecular but not cortical bone microarchitecture in OVX mice similarly to the ASSNAC effect on the trabecular but not the cortical bone.

Based on the observation that ASSNAC exerts its optimal protective effect after 4 weeks followed by some weakening of this effect after 8 weeks of treatment, we assumed that the ASSNAC dose of 50 mg/Kg/day might have been too high. Indeed, the ASSNAC treatment at the lower dose of 20 mg/Kg/day resulted in a more favorable bone outcome in both the femur and vertebra, providing similar protection to that of Alendronate in all vertebra and in the femur full and trabecular but not cortical parameters.

The treatment of OVX mice with ASSNAC in combination with Alendronate enhanced the protective effect of Alendronate on trabecular but not cortical bone microarchitecture in both the femur and the vertebra, which was restored to that of the Sham mice. These results indicate the potential use of ASSNAC in combination with Alendronate; however, further studies aimed at optimizing the ASSNAC and Alendronate doses in order to achieve the protection level of Alendronate treatment but without the side effects associated with the bisphosphonate treatment are needed.

In the present study, both CTX and P1NP serum levels increased 2 weeks after ovariectomy and returned to the basal level after 8 weeks. The increases in CTX and P1NP clearly indicate a significant induction of bone turnover, driven by an increased bone resorption and a partially compensatory increased bone formation, respectively. These results are in agreement with a previous study demonstrating increases in the levels of P1NP and CTX during the first 2 weeks post ovariectomy, returning to their levels in Sham mice after 8 weeks [62]. As expected, Alendronate prevented the OVX-induced bone resorption, keeping CTX at the basal level. Interestingly, ASSNAC treatment alone or in combination with Alendronate modulated the serum levels of both CTX and PINP. Compared to Alendronate alone, the combined Alendronate + ASSNAC treatment resulted in a higher bone mass after 4 weeks, likely due to the deeper inhibition of bone resorption (lower CTX level) during the first weeks following ovariectomy. The effect of ASSNAC on the levels of CTX and P1NP correlates with and strengthens the observed protective effect on bone microarchitecture in OVX mice.

Previous studies demonstrated the contribution of redox imbalance to bone fragility [63] and an increase in oxidative stress biochemical markers in osteoporosis [64,65]. A reduced antioxidant level was found to be associated with increased bone resorption [66], whereas protection against osteoporosis in aged and OVX rats was linked to a reduction in oxidative stress [67].

Our current data showing that the treatment of OVX mice with Alendronate reduces the level of glutathione and increases the level of MDA are in agreement with previous observations in that bisphosphonates induce oxidative stress [24,25]. These data might suggest that Alendronate is not the optimal treatment for osteoporosis, preventing bone resorption on one hand, while causing oxidative stress on the other hand. A previous study suggested that bisphosphonates induce a massive production of ROS in human periodontal ligament fibroblasts, thus contributing to bisphosphonate-related osteonecrosis of the jaw [68]. The oxidative damage might explain, at least partially, the deleterious effect of Alendronate causing atypical femur bone fractures and jaw necrosis [69]. Importantly, our data demonstrate that the treatment of OVX mice with ASSNAC in combination with Alendronate prevents the oxidative damage induced by Alendronate as demonstrated by the attenuation of the ovariectomy-induced GSH decreases and MDA increases. Indeed, these data suggest that the combined treatment of Alendronate and ASSNAC exerts a better protective effect on both femur and vertebra microarchitecture in OVX mice. The beneficial contribution of ASSNAC to bone microarchitecture and its protective effect against oxidative damage advocate for combining ASSNAC with Alendronate to improve bone mass and limit the risk of osteonecrosis.

However, to further establish the potential role of ASSNAC in the treatment of osteoporosis, future studies are required, aimed at the optimization of the ASSNAC dose as a solo treatment as well as the doses of ASSNAC and Alendronate in the combined treatment. These optimizations may enable us to reduce the Alendronate dose and thus prevent its deleterious side effects, without affecting or even improving its anti-osteoporosis effect. Furthermore, studies comparing the effects of ASSNAC alone or in combination with Alendronate to those of other antioxidants are needed as well.

## 5. Conclusions

The current data suggest that a solo treatment with ASSNAC, in view of its antioxidant activity, might play a significant role in the prevention of osteoporosis. Furthermore, ASSNAC treatment in combination with Alendronate may attenuate the Alendronate-associated oxidative stress at the BM milieu and thereby improve its anti-osteoporosis activity. In addition, the antioxidant activity of ASSNAC might also prevent the Alendronate deleterious side effects, such as atypical femur bone fracture and jaw osteonecrosis, both associated with oxidative stress.

## Data Availability

Raw data will be made available upon written request to the corresponding author.
